# Machine learning–based validation of Human Phenotype Ontology (HPO) terms for systemic autoinflammatory diseases using the Eurofever Registry: the ODINO project

**DOI:** 10.3389/fimmu.2026.1936099

**Published:** 2026-10-12

**Authors:** Caterina Matucci-Cerinic, Giacomo Cavalca, Matteo Vergani, Roberta Caorsi, Marielle van Gjin, Guilaine Boursier, Eugenia Mosci, Giovanni Fiorito, Paolo Uva, Marco Gattorno

**Affiliations:** 1 Rheumatology and Autoinflammatory Diseases, IRCCS Istituto Giannina Gaslini, Genoa, Italy; 2 Clinical Bioinformatics Unit, IRCCS Istituto Giannina Gaslini, Genoa, Italy; 3 Department of Pharmacy and Biotechnology, University of Bologna, Bologna, Italy; 4 Department of Genetics, University of Groningen, University Medical Center Groningen, Groningen, Netherlands; 5 Department of Molecular Genetics and Cytogenomics, CHU Montpellier, CeRéMAIA, IRMB, Univ Montpellier, INSERM, Montpellier, France; 6 Gaslini Trial Center, IRCCS Istituto Giannina Gaslini, Genova, Italy

**Keywords:** artificial intelligence, Hpo, human phenotype ontology, machine learning, systemic autoinflammatory diseases

## Abstract

**Background:**

The Human Phenotype Ontology (HPO) terms for systemic autoinflammatory diseases (SAIDs) were revised in 2022, but their diagnostic accuracy has not yet been validated in real-life cohorts.

**Objectives:**

To evaluate the diagnostic accuracy of HPO terms in a real-life cohort of patients enrolled in a large international registry for SAIDs through a machine learning (ML) approach.

**Methods:**

Clinical and genetic data were extracted from the Eurofever Registry (EF) for 2,576 patients. Due to limited sample sizes for rarer conditions, analyses focused on FMF, MKD, TRAPS, CAPS and PFAPA. The 223 clinical EF variables were codified into HPO terms, including terms with missing HPO correspondence. Supervised ML models (Elastic Net, k-Nearest Neighbors, Random Forest, and XGBoost) were trained to classify the five diseases using both multiclass and one-vs-rest (OVR) strategies.

**Results:**

A full HPO correspondence was found for 185 EF terms. Clinically relevant variables frequently missing from HPO, including “ancestry” and “fever length”, were incorporated into the final models. On the held-out test set, the XGBoost OVR framework achieved the following recall values: 87.2% for FMF, 90.4% for PFAPA, 81.1% for CAPS, 55.7% for TRAPS, and 55.6% for MKD. Feature importance analyses highlighted “fever length” as key discriminative variable. Error analysis revealed an enrichment of patients carrying the R92Q TNFRSF1A variant among false-negative predictions for TRAPS. Unsupervised analysis showed no clear separation between FMF patients carrying one versus two pathogenic *MEFV* mutations.

**Conclusions:**

HPO-based phenotypic modeling supports accurate classification of the 5 most frequent recurrent fevers included in this study. The HPO database should be enriched by including the missing terms, especially “fever length”, “ancestry”, and the symptoms frequencies according to Eurofever. An ontology-driven diagnostic tool applicable to both clinical research and computational settings for recurrent fevers is now available.

## 1 Introduction

Systemic autoinflammatory diseases (SAIDs) are characterized by a sudden, antigen-independent over-activation of the immune response leading to an exaggerated sterile inflammatory reaction. This group defines a new class of diseases for which more than 100 associated genes have been identified over the past 20 years (1), and thanks to the remarkable advancement of genetic sequencing, the number of newly discovered genes continues to grow. The first SAIDs to be identified were the so-called recurrent fever syndromes: Familial Mediterranean Fever (FMF), Mevalonate Kinase Deficiency (MKD) or hyper-IgD syndrome, TNF Receptor-Associated Periodic Syndrome (TRAPS), and Cryopyrin-Associated Periodic Syndromes (CAPS). Over the following years, new diseases were discovered, not necessarily characterized by recurrent fever, but still characterized by an inflammasome hyperactivation with overproduction of interleukin-1 (IL-1), and recently termed “inflammasomopathies” (2). The clinical phenotype of SAIDs is therefore quite heterogeneous, involving multiple organs, and possibly overlapping with other monogenic and multifactorial autoinflammatory diseases, as well as autoimmune diseases. Moreover, incomplete penetrance and the identification of variants of unknown significance (VUS) further complicate genotype–phenotype correlations. As a result, many patients experience significant diagnostic delay, with potential consequences for disease management, long-term outcomes, and quality of life.

International registries such as Eurofever (EF) have been established to collect clinical characteristics, complications, and treatment responses of these diseases, with currently more than 5,000 patients with different SAIDs recorded (3, 4). Based on the statistical analysis of data cataloged in EF, evidence-based classification criteria have been developed for hereditary recurrent fevers (FMF, MKD, TRAPS, CAPS) (5). Moreover, a registry dedicated to all known genetic variants associated with SAIDs has been created, the INFEVERS registry (6, 7). This database is continuously updated and includes also VUS reported in scientific studies.

Even with these tools—considering both clinical and genetic variables—the diagnosis of SAIDs remains a challenge. Genetic variants are not always easy to interpret, raising the issue of correctly contextualizing genetic findings in diseases with inflammatory phenotypes.

Therefore, new diagnostic strategies have been developed, progressing towards the use of standardized, computerized terminology that allows for the description of phenotypic characteristics—crucial for genotype–phenotype correlation analysis. The Human Phenotype Ontology (HPO) was developed to address this gap by providing a standardized, hierarchical vocabulary for phenotypic abnormalities (8). HPO is a terminology that enables consistent annotation of clinical features and supports interoperability across datasets, registries, and analytical tools. The system is hierarchically organized with tree-like structures that allow connection of related terms and phenotypes. Each disease with a known genetic cause is linked to the corresponding HPO terms describing its clinical features. This allows researchers to suggest possible candidate genes based on a set of HPO terms. The HPO system revolutionized patient genetic analysis and now underpins several international initiatives, including SOLVE-RD, RD-CONNECT, the 100K Genome Project, the NIH Undiagnosed Diseases Program, and others. A key feature of the HPO system is its ability to interconnect with other standard ontologies. This interoperability is leveraged by various algorithms (e.g., Exomiser, LIRICAL) to identify potential disease-causing variants. Its increasing adoption in rare disease research has facilitated phenotype-driven gene discovery and computational diagnostics.

In 2021, a group of Experts revised and updated the HPO terminology for 73 inborn errors of immunity, including SAIDs (9, 10). However, the accuracy of the new and revised HPO terms in correctly classifying SAIDs has not yet been validated in a cohort of real patients.

In parallel with advances in phenotypic standardization, machine learning (ML) approaches have emerged as powerful tools for analyzing complex, high-dimensional clinical data. Rather than relying on single pathognomonic features, ML methods can identify complex patterns of variables associated with specific diseases, that may not be readily recognized through conventional clinical reasoning. This data-driven approach has shown potential in supporting diagnostic processes, particularly in heterogeneous conditions, by integrating multiple clinical and genetic features to improve pattern recognition and disease classification (11–13). Its application in rare diseases remains constrained by small sample sizes, underscoring the need for high-quality, standardized datasets.

The ODINO project (Optimization of the Diagnostic approach for INborn errors of immunity leading to hyper-inflammatiOn) was established to improve the diagnostic approach to Inborn Errors of Immunity (IEI) using SAIDs as proof of concept. The present study addresses one of the main objectives of the ODINO initiative. Specifically, the aim of this project is to validate the diagnostic accuracy of HPO terms in a real-life cohort of patients with SAIDs through a ML approach and to implement the HPO terminology within the EF registry.

## 2 Methods

### 2.1 HPO encoding and patient extraction

The clinical variables included in EF were systematically mapped to HPO terms, and missing or partially represented terms were identified.

From EF, all patients with monogenic SAIDs were initially selected (3,040 patients with 18 monogenic SAIDs). As among polygenic recurrent fevers Periodic Fever, Aphthous Stomatitis, Pharyngitis, Adenitis (PFAPA) is the most frequent disease, we decided to include it in the analysis as confounder (n=642). The main characteristics of the EF Registry have been previously described (3, 4). Data in EF are encrypted, and patients are identifiable only by the enrolling center. Each participating center obtained approval from its local Ethics Committee for data entry. For all patients clinical and genetic data were extracted. All the patients were screened for missing/non-clear clinical data. If needed, the enrolling centers were contacted to fill in the missing information. When possible, missing data were re-integrated into the system, otherwise patients were excluded from the analysis. Patients’ mutational profile was thoroughly screened to confirm the diagnosis.

### 2.2 Comparison of terms’ frequencies in HPO and Eurofever

In the HPO system, each disease is characterized by a list of symptoms, each associated with a frequency extrapolated from the literature. To evaluate whether the symptoms’ frequencies reported on the HPO website were consistent with those observed in the EF cohort, a Uniform Manifold Approximation and Projection (UMAP) was used to project disease profiles into a two-dimensional space for comparison (14). Each disease annotated in HPO was represented as a vector of HPO terms weighted by the reported frequencies. When frequencies were not reported as numerical values but as categorical annotations, the midpoint of the corresponding frequency interval was used: Frequent (79-30%) = 55, Occasional (29%-5%) = 17.5, Very rare (<4%-1%) = 2.5. These disease profiles were then projected into a two-dimensional space using UMAP. In this representation, diseases located close to each other share similar patterns of HPO term frequencies. Each EF patient was finally projected onto the same UMAP space based on the observed HPO-coded symptoms, to evaluate possible overlap between EF patients and the HPO disease profiles.

### 2.3 Patients’ selection for machine learning analysis

From EF, 3,682 patients with 18 monogenic SAIDs and a multifactorial confounding disease such as PFAPA were initially identified. However, due to the limited number of patients for several rare conditions (e.g., PAPA syndrome, n=35; Majeed syndrome, n=5; deficiency of IL-1 receptor antagonist, n=3), ML analyses were restricted to diseases with larger sample sizes (N > 200), leading to the inclusion of only monogenic recurrent fevers (FMF, MKD, TRAPS, CAPS) and PFAPA. Among monogenic diseases, only patients with a confirmatory genotype according to the classification criteria for recurrent fevers (5) were included, except for TRAPS, for which patients carrying the low-penetrance variant R92Q were also included in the analysis if they met the clinical classification criteria. A separate sub-analysis was later conducted in these patients. For FMF, also monoallelic patients (carrying one pathogenic mutation with a corresponding clinical phenotype) who were classified as FMF according to the EF classification criteria were retained for the analysis, and later compared with biallelic patients (homozygous or compound heterozygous). This approach led to the final inclusion of 1,966 patients with monogenic recurrent fevers. For PFAPA, only patients who met the EF classification criteria were included (610 patients). The final cohort included 2,576 patients with FMF (n=1,182), CAPS (n=303), TRAPS (n=277), MKD (n=204), and PFAPA (n=610) (**Table 1**). In **Figure 1** the flow-chart of the whole selection process is reported.

**Table 1:** Descriptive characteristics of the dataset.

	FMF (n=1182)	PFAPA (n=610)	CAPS (N = 303)	TRAPS (N = 277)	MKD (N = 204)
# of Females (%)	563 (48)	263 (43)	153 (50)	136 (49)	107 (52)
Age at onset (years), median (Q1-Q3)	3.53 (1.64-7.43)	1.83 (0.99-3.22)	1.81 (0.19-7.84)	4.38 (1.00-13.83)	0.70 (0.19-2.54)
Fever length(days), median (Q1-Q3)	2 (2-3)	4 (3-5)	0 (0-3))	6 (3-13)	4 (3-6)

CAPS, Cryopyrin-Associated Periodic Syndromes; FMF, Familial Mediterranean Fever; MKD, Mevalonate Kinase Deficiency; PFAPA, Periodic Fever, Aphthous Stomatitis, Pharyngitis, Adenitis; TRAPS, TNF Receptor-Associated Periodic Syndrome.

**Figure 1:**
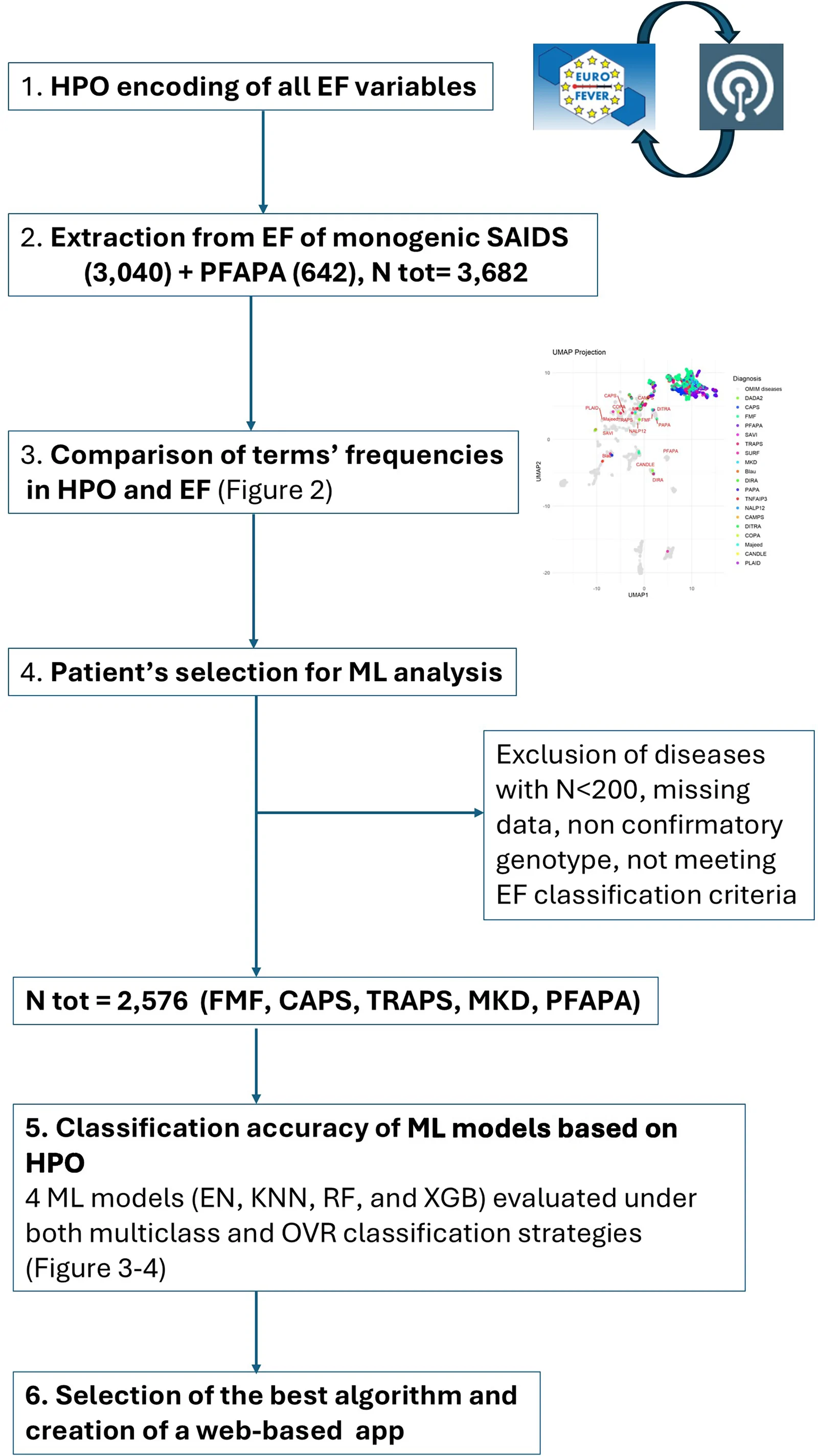
Flow-chart of variables’ encoding, patient’s selection process and methodological outline.

After the encoding of all EF variables, the included patients were reannotated using standardized HPO descriptors, together with key demographic and clinical variables. EF non-informative “other” categories or variables not present in any of the included patients were excluded from the analysis.

### 2.4 Classification accuracy of HPO terms using machine learning models

Supervised ML models were developed to evaluate the diagnostic performance of HPO-based phenotyping, in addition to gender, age at onset, ancestry, and fever length. To better exploit the hierarchical structure of the HPO, each annotated term was propagated to its ancestor terms within the ontology, and the resulting extended dataset was used for model development. A sensitivity analysis was then performed using the original non-propagated dataset to assess the effect of term propagation on model performance (see **Supplementary Methods** for details).

Four algorithms were evaluated: Elastic Net (EN), K-Nearest Neighbors (KNN), Random Forest (RF), and Extreme Gradient Boosting (XGB). Two classification strategies were investigated: a multiclass approach, in which patients were directly assigned to one of the diagnostic categories, and a one-vs-rest (OVR) approach, in which disease-specific binary classifiers were trained for each condition. The dataset was randomly divided into training (70%) and test (30%) sets, preserving the diagnoses distribution across the two subsets. Model training and hyperparameter tuning were performed on the training set using nested cross-validation (CV), with the F2-score used as the primary optimization metric to prioritize recall. Model performance was evaluated on the independent test set using F2-score and recall. SHAP feature importance and error analyses were performed to identify clinically relevant variables and patterns of misclassification.

In the OVR framework, each disease-specific classifier generated an independent binary probability. To obtain a single disease prediction for each patient, the five OVR probabilities were subsequently combined by dividing each disease-specific probability by the sum of the five probabilities for that patient, and the disease with the highest normalized score was assigned as the final prediction. This normalization preserves the relative ranking of the original OVR probabilities and yields scores summing to one, enabling a single-label evaluation directly comparable with the multiclass framework (see **Supplementary Methods**).

In addition, UMAP was used to visualize phenotypic similarity between FMF patients with one versus two pathogenic *MEFV* mutations. To quantitatively assess group separability, the silhouette score was calculated in the original feature space using the same HPO and clinical/demographic variables. Age at onset and fever length were standardized, while gender and ancestry were one-hot encoded.

Finally, the best-performing model was implemented in a web-based application to provide disease probability estimates based on HPO inputs. Detailed descriptions of data preprocessing, model optimization, and statistical analyses are provided in the **Supplementary Methods**.

## 3 Results

### 3.1 Encoding of clinical variables according to HPO terms

A total of 223 EF variables (207 clinical, 12 laboratory, 4 demographic) were mapped to HPO codes. Of these, 185 showed full correspondence, 26 partial correspondence (for 6 terms more than 1 HPO corresponding to 1 EF term), and 13 had no corresponding HPO term. Among these 13 terms, clinically relevant variables such as “age at onset” and “fever length” were present in all patients. Age at onset showed partial correspondence, being represented by multiple categorical HPO terms (e.g., neonatal, pediatric, adult onset). Additionally, redundancy among 5 EF variables was identified (**Supplementary Table 1**).

After data preprocessing, involving removal of terms that were not present in any patient, 168 HPO terms and 4 demographic/clinical variables (gender, age at onset, ancestry, and fever length) were retained, yielding a total of 172 predictors. EF terms with an absolute, partial or absent HPO correspondence are reported in **Supplementary Tables 1–3**. Following hierarchical propagation of HPO terms, the number of HPO-derived features increased to 429, while the same set of clinical and demographic variables was retained.

During the mapping process, structural limitations within the HPO system were identified. Specifically, disease annotations differed between OMIM and ORPHA entries, which include distinct sets of HPO terms. Moreover, symptom frequencies are inconsistently reported—either as absolute values (e.g., 1/100) in OMIM or as qualitative categories (e.g., “frequent”) in ORPHA—limiting comparability. In addition, numerical frequencies are often derived from small cohorts and may not reflect real-life prevalence.

### 3.2 Comparison of terms’ frequencies in HPO and Eurofever

UMAP analysis (**Figure 2**) showed a clear separation between EF patients and disease clusters derived from HPO annotations, indicating substantial differences in terms’ frequencies between the EF cohort and the HPO database. Although several HPO terms were shared between the two systems, their reported frequencies often differed markedly. In addition, a considerable number of clinical features were exclusively present in EF, in some cases with relatively high prevalence despite being absent from the HPO-based disease annotations. Based on these findings, the development of a diagnostic support tool for SAIDs was motivated, with the model trained on HPO term frequencies observed in the EF dataset rather than those reported in HPO.

**Figure 2:**
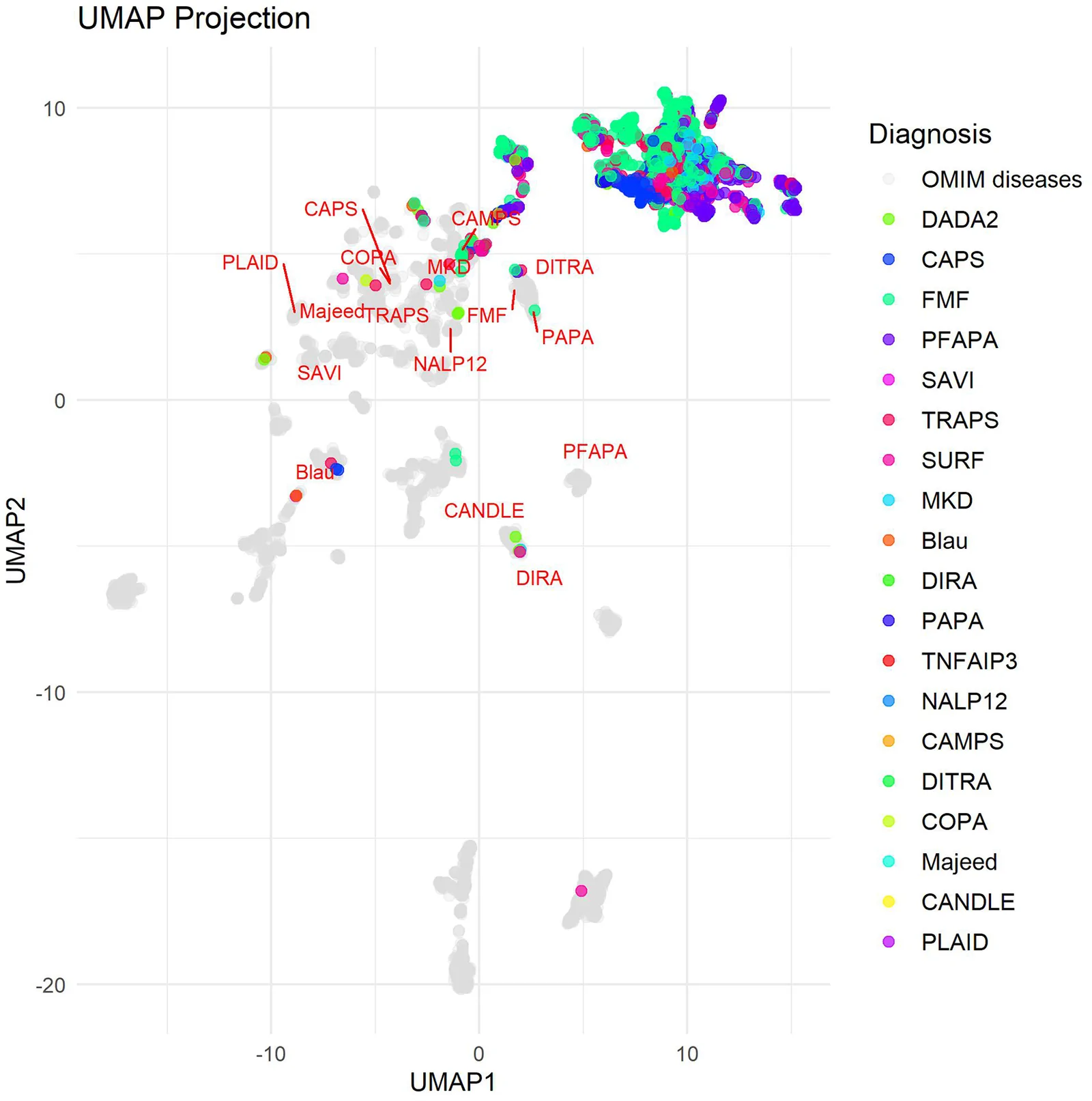
Two-dimensional UMAP representation of disease profiles derived from HPO annotations (labeled in red) and individual patients from the Eurofever (EF) cohort (colored points). Each EF patient is projected based on the presence of HPO-coded clinical features, while HPO disease profiles are constructed using term frequencies reported in the ontology. A clear separation is observed between EF patients and HPO-derived disease clusters, highlighting differences in symptom frequency distributions between real-world data and HPO annotations.

### 3.3 Evaluation of HPO terms performance using machine learning models

As specified in the Methods, the following approach was limited to the diseases with the highest numerosity, monogenic recurrent fevers (FMF, MKD, TRAPS, CAPS) and their main multifactorial confounder, PFAPA.

#### 3.3.1 Models’ comparison and selection

Four ML models (EN, KNN, RF, and XGB) were evaluated under both multiclass and one-vs-rest (OVR) classification strategies. Model comparison and selection were based on performance across the 5 CV folds within the training set. Overall, the OVR approach achieved higher predictive performance than the multiclass framework for most diseases, with the exception of MKD, for which multiclass performance was slightly superior (**Table 2**; **Supplementary Figure 1**).

**Table 2:** Comparison of the best-performing models for each disease under Multiclass and One-vs-Rest (OVR) classification strategies, based on 5-fold CV performance in the training set.

Disease	Best model (Multiclass)	F2-score Multiclass	Recall Multiclass	Best model (OVR)	F2-score OVR	Recall OVR
CAPS	EN	0.864 ± 0.028	0.879 ± 0.039	EN	**0.877 ± 0.037**	**0.909 ± 0.047**
FMF	XGB	0.849 ± 0.019	0.834 ± 0.015	EN	**0.866 ± 0.018**	**0.866 ± 0.022**
MKD	XGB	**0.639 ± 0.105**	0.667 ± 0.111	XGB	0.62 ± 0.057	0.68 ± 0.077
PFAPA	XGB	0.836 ± 0.029	0.848 ± 0.031	XGB	**0.858 ± 0.022**	**0.886 ± 0.019**
TRAPS	XGB	0.581 ± 0.085	0.587 ± 0.105	XGB	**0.626 ± 0.034**	**0.687 ± 0.039**

Performance is reported as the mean ± standard deviation of the F2-score and recall across the five cross-validation folds. For each disease, the best performance achieved between the two classification strategies is highlighted in bold.

Within the OVR framework, XGB achieved the highest F2-score for MKD, PFAPA, and TRAPS, whereas EN showed slightly higher performance for CAPS (0.877 vs 0.858) and FMF (0.866 vs 0.861). Conversely, XGB showed a larger advantage over EN for MKD (0.620 vs 0.580) and TRAPS (0.626 vs 0.569), the two diseases with the lowest overall classification performance. When averaged across the five disease-specific OVR classifiers, XGB achieved the highest mean F2-score (0.764), compared with EN (0.750). Therefore, considering its more balanced performance across diseases and the advantage of using a common algorithm for all disease-specific classifiers, XGB was selected as the modeling algorithm for the OVR framework. Detailed cross-validation performance metrics for all models are reported in the Excel **Supplementary Material Tables E1–E4**.

On the held-out test set, the XGB OVR framework showed a slightly higher overall F2-score compared with the multiclass XGB model (0.74 vs 0.73).

The disease-specific OVR classifiers were first evaluated independently at a probability threshold of 0.50 (**Figure 3B**). To derive the final single-label OVR prediction, the five disease-specific outputs were then combined and the disease with the highest normalized score was assigned to each patient (**Figure 3C**). This final representation was used for comparison with the multiclass framework and for reporting the main performance metrics.

**Figure 3:**
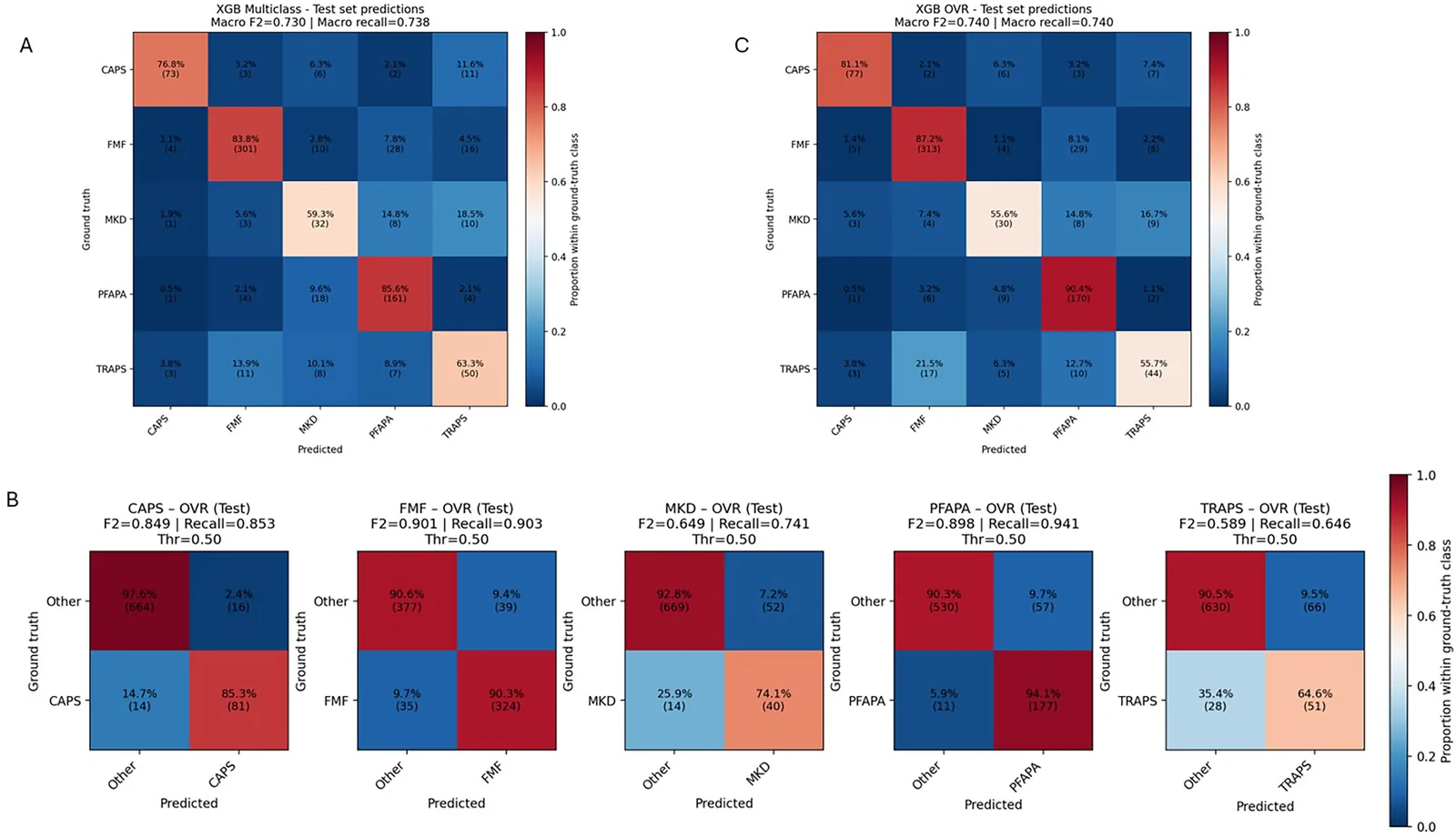
**(A)** Confusion matrix showing results of the multiclass classification analysis on the test set; **(B)** confusion matrices obtained by evaluating each disease-specific XGB OVR classifier independently on the test set using a probability threshold of 0.50. **(C)** Final single-label OVR classification performance on the test set. For each patient, the five disease-specific OVR probabilities were divided by their sum and the disease with the highest normalized score was assigned as the predicted diagnosis.

Notably, recall improved in OVR classification for CAPS (76.8% to 81.1%), FMF (83.8% to 87.2%), and PFAPA (85.6% to 90.4%), whereas it decreased for MKD (59.3% to 55.6%) and TRAPS (63.3% to 55.7%) (**Figure 3**; **Supplementary Table 4**). Based on these results, XGB combined with the OVR strategy was selected as the final modeling framework for subsequent analyses. Sensitivity analyses performed using the non-propagated dataset showed comparable predictive performance, indicating that propagation of HPO terms to ancestor nodes did not substantially affect classification accuracy. Patient-level predicted probabilities for the test set are reported in Excel **Supplementary Tables E5 and E6**.

#### 3.3.2 Probability prediction

To further characterize model performance, we analyzed the distribution of predicted probabilities for true positives and false positives across diseases. For most conditions, predicted probabilities were significantly higher for correct classifications (true positives) than for incorrect ones (false positives) (p < 0.05 for all diseases except MKD) (**Figure 4A**), indicating that the model assigns higher confidence to correct predictions, whereas misclassifications are generally associated with lower confidence scores.

**Figure 4:**
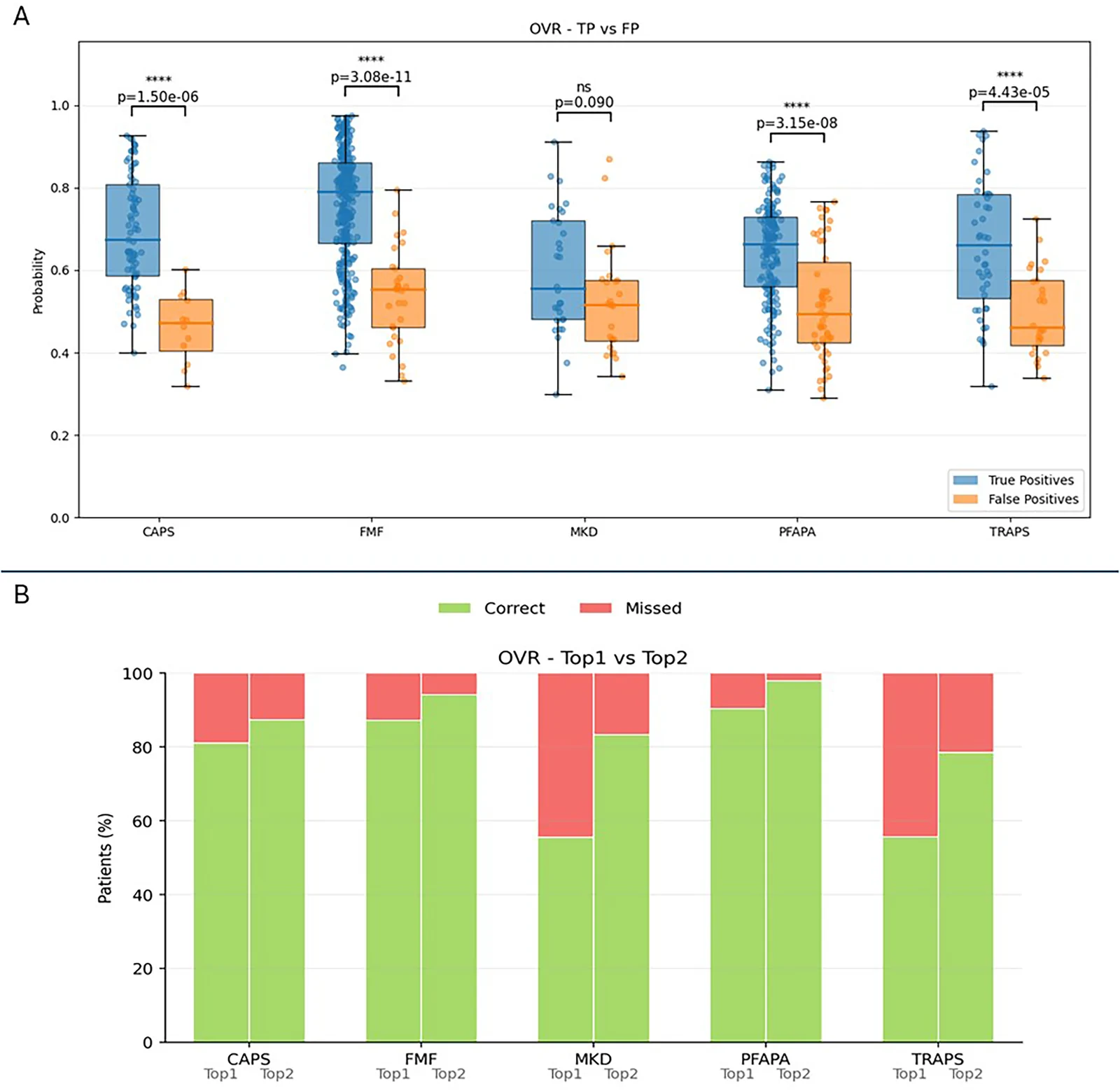
**(A)** Distribution of predicted probabilities for correct and incorrect classifications. For each disease, predicted probabilities associated with true positive (TP) and false positive (FP) predictions are shown. Correct classifications are associated with significantly higher probability values compared with incorrect predictions for all diseases except MKD, indicating that prediction confidence is strongly associated with classification accuracy. **(B)** Diagnostic performance considering the two most probable predicted diseases (top-2 predictions). For each disease, the proportion of correctly identified cases increases when the two highest predicted probabilities are considered instead of the top prediction alone, indicating that the correct diagnosis is frequently captured among the two most probable model predictions.

Based on this, we evaluated whether the correct diagnosis was included among the two most probable predictions (top-2 analysis). In a substantial proportion of misclassified cases, the true diagnosis corresponded to the second highest predicted probability, suggesting that the model frequently identifies the correct disease among the two leading diagnostic candidates even when the top prediction is incorrect. Detailed results are shown in **Figure 4B** and Excel **Supplementary Tables E7 and E8**.

#### 3.3.3 Feature importance

Feature importance analysis based on SHAP values was used to quantify the contribution of each variable to disease classification. This approach allows estimation of how much each feature influences the model’s prediction. Several variables were found to consistently contribute to disease discrimination across models. In particular, “age at onset” and “fever length” were identified as key predictors across multiple diseases. Disease-specific rankings are shown in **Supplementary Figure 2** and reported in Excel **Supplementary Tables E9–13**.

#### 3.3.4 Comparison between TRAPS with pathogenic variants and individuals carrying the R92Q variant

Patients carrying the R92Q variant were initially included in the TRAPS group when fulfilling EF clinical criteria. Given the lower classification performance observed for TRAPS, in order to evaluate a possible causality with the inclusion of R92Q patients in this group, a subgroup analysis was performed. R92Q patients were more frequently represented among misclassified cases compared with correctly classified cases (Chi-squared test p = 0.062; **Figure 5**). Specifically, 21/37 (57%) of R92Q were classified in the “false negative” group, while 16/37 (43%) in the true positive group.

**Figure 5:**
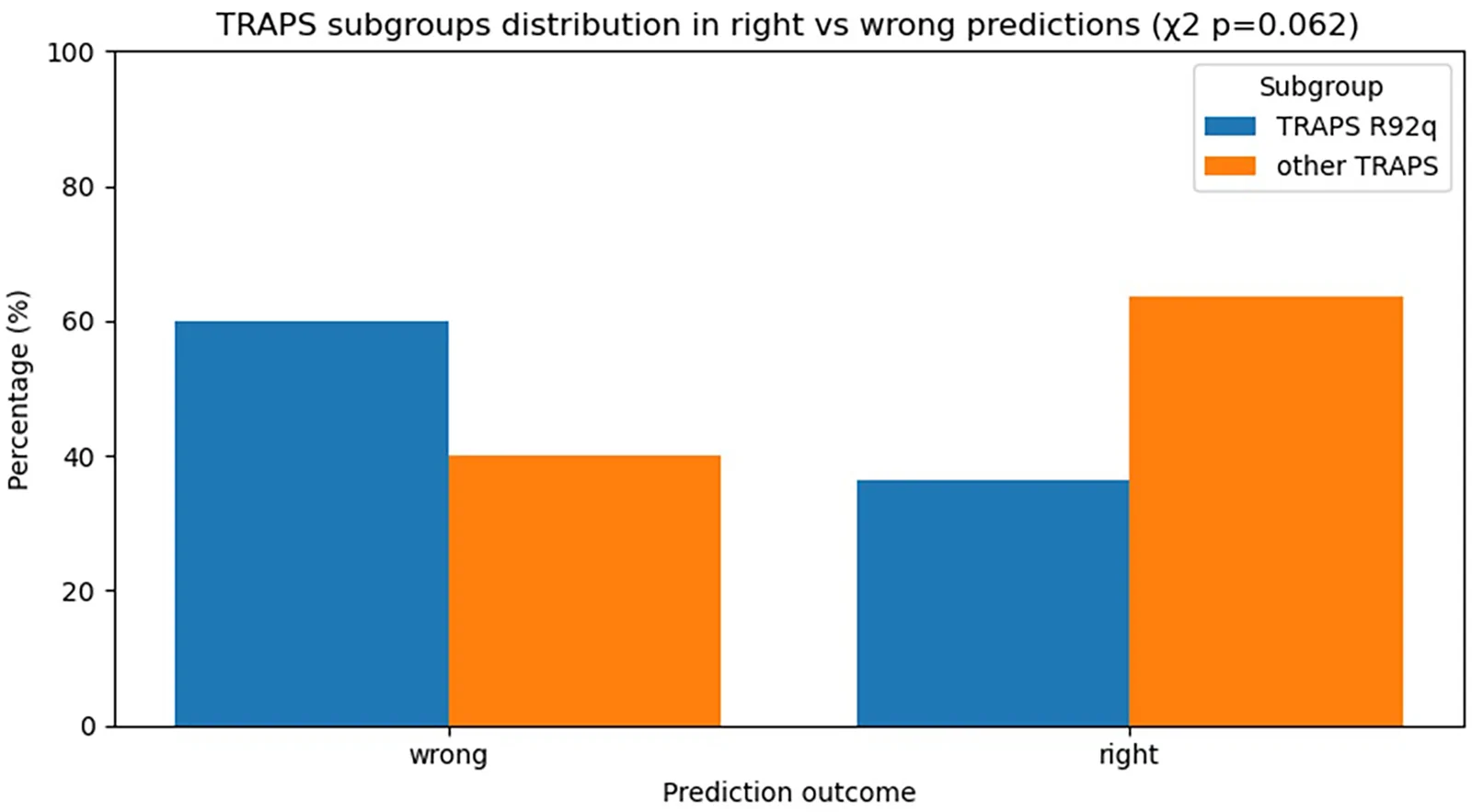
Error analysis of TRAPS predictions. Distribution of correctly and incorrectly classified TRAPS patients according to genotype. An enrichment of patients carrying the R92Q variant is observed among misclassified cases (Chi-squared test, p = 0.062).

#### 3.3.5 Comparison between monoallelic *versus* biallelic FMF patients

In the FMF group, both monoallelic (one pathogenic mutation who fulfilled the EF classification criteria) and biallelic patients (homozygous or compound heterozygous) were included. UMAP visualization showed substantial overlap between the two groups (**Figure 6**). This observation was supported by a silhouette score of 0.010 calculated in the original feature space, indicating very limited phenotypic separability between monoallelic and biallelic FMF patients based on the analyzed HPO and clinical features.

**Figure 6:**
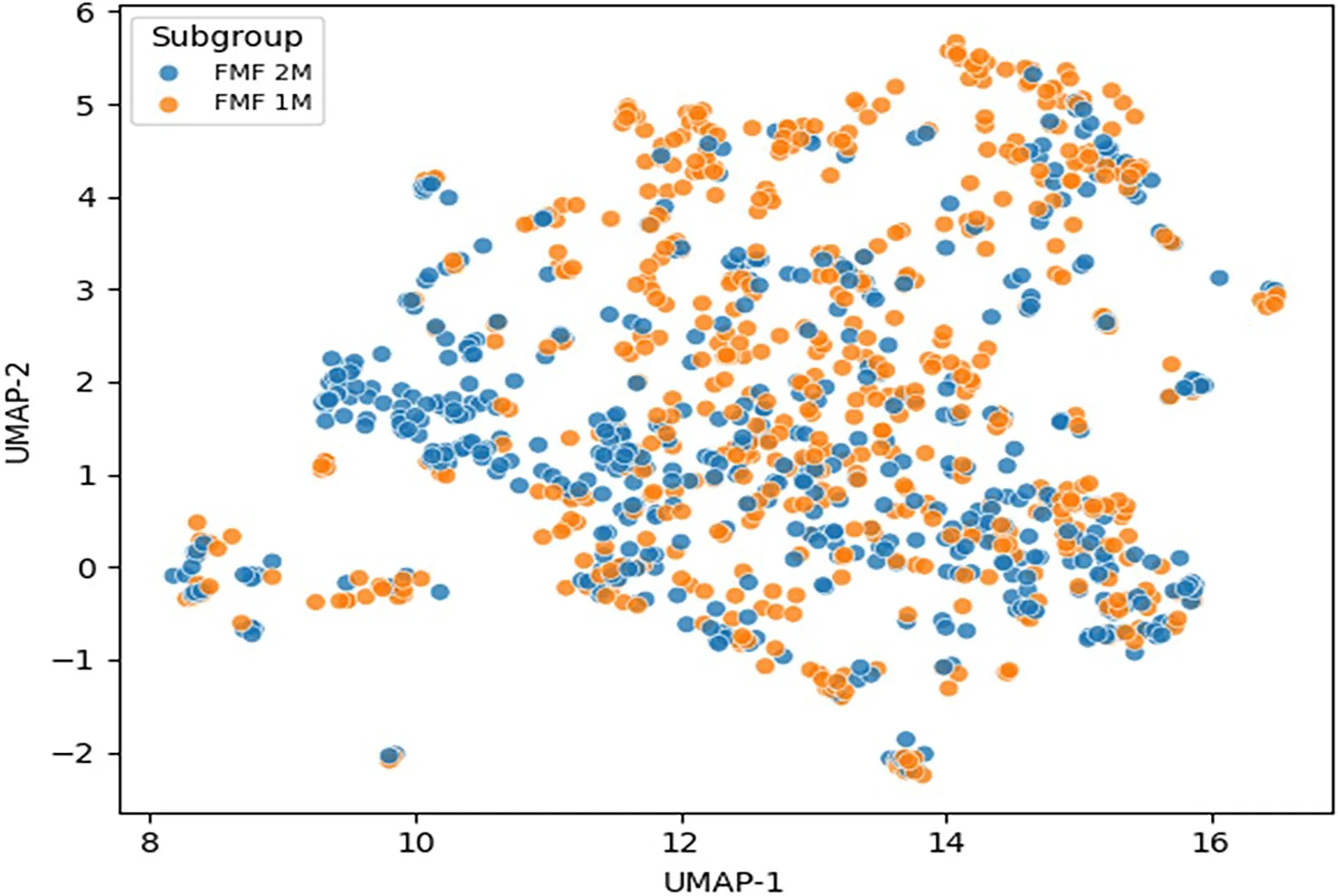
Phenotypic distribution of monoallelic and biallelic FMF patients. UMAP projection of FMF patients based on HPO and clinical features, stratified according to the presence of one (monoallelic, 1M) or two (biallelic, 2M) pathogenic MEFV mutations. The two groups show substantial overlap, Quantitative assessment in the original feature space yielded a silhouette score of 0.010, indicating a very limited group separability.

### 3.4 Creation of a web-based app

The final XGB OVR model was integrated into an interactive Streamlit-based web application. Clinicians can access the application when needed, complete a structured form by entering clinical features and HPO terms describing an individual patient, and obtain disease probability scores across the five target conditions. The application is publicly available at https://biodaas.gaslini.org/hpo-dx/, and the source code can be accessed at https://github.com/Jackcava/hpo-dx. This tool provides an intuitive proof-of-concept application for translating the proposed ML framework into a clinically oriented diagnostic support system, providing practical assistance to geneticists and rheumatologists in the correct interpretation of patients’ phenotype.

## 4 Discussion

This study systematically linked Eurofever clinical variables to HPO terms for the main Autoinflammatory diseases and evaluated their diagnostic performance in a real-world cohort of patients with recurrent fevers using a ML approach. In parallel, we developed an ontology-driven diagnostic tool applicable to both clinical research and computational settings.

The first major achievement is the systematic coding of Eurofever clinical variables into HPO terms. The majority of Eurofever variables could be successfully encoded within the HPO framework, supporting its overall adequacy in describing periodic fever syndromes. However, this process also highlighted several critical gaps with direct clinical and computational implications. Notably, key variables for the differential diagnosis of recurrent fevers, namely “fever length” and “ancestry”, are not adequately represented in HPO. “Fever length” could be incorporated into the HPO terminology as a clinically relevant descriptor, whereas “ancestry”, although outside the scope of HPO, should be considered in classification due to its epidemiological relevance. These variables are consistently recorded in Eurofever and are included in the classification criteria for recurrent fevers (5). Importantly, these features also emerged as highly informative predictors in our ML analyses. In particular, “fever length” was among the most discriminative variables across diseases, with opposite contributions reflecting well-known clinical differences: shorter episodes in FMF and longer inflammatory attacks in TRAPS. Similarly, “ancestry” contributed significantly to disease classification, consistent with the recognized population distribution of these conditions, especially FMF.

Beyond missing terms, this work also identified structural limitations within the HPO system. Differences between OMIM- and ORPHA-based annotations, as well as inconsistencies in how symptom frequencies are reported, limit comparability and clinical usability. In particular, frequency estimates derived from small cohorts may not reflect real-world disease expression. Integrating registry-based frequencies, such as those derived from Eurofever, may improve both the clinical accuracy and computational utility of HPO annotations.

The supervised ML analyses demonstrated that HPO-derived phenotypic representations have strong diagnostic potential. While the OVR strategy showed only a modest overall performance advantage compared with the multiclass approach, it offers a relevant methodological advantage by allowing to model each disease independently through disease-specific classifiers. This enables a more detailed investigation of disease-specific predictive patterns and facilitates model interpretation through the extraction of feature importance profiles for each condition. The effect of the OVR strategy, however, differed across diseases. The improved recall observed for CAPS, FMF and PFAPA may reflect their larger sample sizes and relatively distinctive phenotypic profiles, which may particularly benefit from disease-specific binary classification. Conversely, the lower final recall observed for MKD and TRAPS may be related to their smaller sample sizes and greater phenotypic heterogeneity and overlap with other recurrent fever syndromes. Notably, the individual OVR classifiers achieved higher recall for MKD and TRAPS than the final combined OVR output, suggesting that part of this loss occurs when the five disease-specific predictions compete in the final single-label classification.

Further insights into model behavior emerged from the analysis of predicted probabilities. Correct classifications (true positives) were associated with significantly higher predicted probabilities than incorrect ones (false positives), indicating that the model tends to assign higher confidence scores to accurate diagnoses and may help guide clinical interpretation. At the same time, this finding suggests that predictions associated with relatively low probability values should be interpreted with caution. In this context, considering the two most probable predicted diseases may represent a clinically useful strategy. When the top two predictions were taken into account, diagnostic accuracy increased substantially. A substantial proportion of misclassified cases corresponded to the second-ranked prediction, especially for more challenging conditions such as MKD and TRAPS. This finding supports the potential utility of ranked diagnostic outputs in clinical decision-support tools, particularly in cases with overlapping phenotypes.

Model comparison showed that no single algorithm was uniformly optimal across all diseases. In the OVR framework, EN slightly outperformed XGB for CAPS and FMF, whereas XGB achieved the highest F2-score for MKD, PFAPA, and TRAPS and the highest mean F2-score across the five disease-specific classifiers. Importantly, the advantage of XGB over EN was more pronounced for MKD and TRAPS, which represented the most challenging diseases to classify. For this reason, XGB was selected as a common modeling algorithm, providing a consistent framework across all disease-specific classifiers while maintaining a balanced overall performance.

The good performance of XGB may partly reflect its ability to capture non-linear relationships and interactions among multiple phenotypic features, which may be particularly relevant in clinically heterogeneous diseases. However, the present study was not designed to formally investigate the reasons underlying differences in performance between algorithms, and this interpretation should therefore remain hypothesis-generating.

The OVR strategy showed only a modest overall performance advantage compared with the multiclass approach, but it also provided an important methodological advantage by modeling each disease independently. This enabled a more detailed investigation of disease-specific predictive patterns and facilitated interpretation through disease-specific feature importance profiles.

The prioritization of recall through the use of the F2-score proved appropriate in this clinical setting, where minimizing false negatives is essential. Sensitivity analyses further showed that the inclusion of HPO ancestor terms did not significantly improve performance but enhanced interpretability, supporting the use of hierarchical phenotypic representations.

A clinically relevant observation emerged from the error analysis of TRAPS. Patients carrying the R92Q variant were more frequently represented among misclassified cases (false negatives). Although this enrichment did not reach statistical significance (p=0.062), this observation may suggest a potential phenotypic divergence between R92Q-associated disease and classical TRAPS associated with pathogenic TNFRSF1A variants. This result would be in line with the concept that R92Q-associated disease represents a distinct clinical entity or a low-penetrance variant with unique clinical characteristics (15). The higher proportion of R92Q carriers among misclassified patients may also have contributed to the lower classification performance observed for TRAPS and highlights how ML-based approaches can uncover meaningful genotype–phenotype relationships beyond traditional categorical diagnoses.

Another important finding concerns the comparison between monoallelic and biallelic FMF patients. The substantial overlap observed in the UMAP visualization, together with a silhouette score close to zero (0.010), indicates very limited phenotypic separability between the two groups based on the analyzed HPO and clinical features. This observation confirms the recent theories that challenge the traditional view of FMF as a strictly autosomal recessive disorder and supports a disease model in which patients with a single high-penetrance variant and a compatible clinical phenotype can be considered affected as biallelic patients (16–18). Nevertheless, only a minority of individuals carrying a single pathogenic MEFV variant develop FMF, suggesting that additional modifiers are required for disease expression. These findings have important implications not only for disease classification but also for clinical management and genetic counseling.

Several limitations of this paper should be acknowledged. First, the analysis was intentionally restricted to five diseases with sufficient representation in the Eurofever Registry. While this choice ensured robust numbers, it limits the application of the findings to the broader spectrum of SAIDs, many of which remain extremely rare. Extending our work to other SAIDs, beyond recurrent fevers, will be essential to fully exploit its diagnostic potential. Second, although the HPO mapping was systematic and comprehensive, it remains constrained by the current structure and content of the ontology. Some of the most relevant limitations identified—such as missing terms and suboptimal disease-level organization—require community-wide efforts and cannot be fully addressed within a single project. Third, an external validation outside the Eurofever Registry was not performed. However, the use of a sizeable held-out test set, together with the international and multicenter nature of the Eurofever cohort, allowed the model to be evaluated on a heterogeneous population of patients from different centers and countries. We believe that this evaluation framework provides a robust assessment of model performance and makes us confident that the approach may generalize well beyond the training data. Nevertheless, validation in an independent external cohort will be important to formally confirm its generalizability.

In conclusion, this work demonstrates that HPO terms, when applied to large real-life clinical registries, can accurately capture disease-specific patterns and support high-performing diagnostic algorithms for SAIDs. By identifying critical gaps in the current HPO structure and integrating registry-derived data, this work provides both methodological and clinically relevant advances. The developed algorithm is accurate and represents a promising supporting tool that could be extended to other recurrent fevers. Moreover, the integration of standardized HPO terms within Eurofever establishes the Registry as a continuous resource for refining and validating HPO annotations, enabling future extension of this approach to additional diseases currently limited by small sample size.

Future studies should extend this approach to a broader spectrum of SAIDs and contribute to the refinement of HPO disease annotations. Such effort will provide more precise, data-driven diagnosis and a deeper understanding of the heterogeneity of autoinflammatory diseases.

## Data Availability

The raw data supporting the conclusions of this article will be made available by the authors, without undue reservation.

## References

[1] Papa R Picco P Gattorno M . The expanding pathways of autoinflammation: a lesson from the first 100 genes related to autoinflammatory manifestations. Adv Protein Chem Struct Biol. (2020) 120:1–44. doi: 10.1016/bs.apcsb.2019.11.001 32085880

[2] Nigrovic PA Lee PY Hoffman HM . Monogenic autoinflammatory disorders: conceptual overview, phenotype, and clinical approach. J Allergy Clin Immunol. (2020) 146:925–37. doi: 10.1016/j.jaci.2020.08.017 33160483 PMC7727443

[3] Ozen S Frenkel J Ruperto N Gattorno M Eurofever Project . The Eurofever Project: towards better care for autoinflammatory diseases. Eur J Pediatr. (2011) 170:445–52. doi: 10.1007/s00431-011-1411-z 21360011

[4] Toplak N Frenkel J Ozen S Lachmann HJ Woo P Koné-Paut I . An international registry on autoinflammatory diseases: the Eurofever experience. Ann Rheum Dis. (2012) 71:1177–82. doi: 10.1136/annrheumdis-2011-200549 22377804

[5] Gattorno M Hofer M Federici S Vanoni F Bovis F Aksentijevich I . Classification criteria for autoinflammatory recurrent fevers. Ann Rheum Dis. (2019) 78:1025–32. doi: 10.1136/annrheumdis-2019-215048 31018962

[6] Milhavet F Cuisset L Hoffman HM Slim R El-Shanti H Aksentijevich I . The infevers autoinflammatory mutation online registry: update with new genes and functions. Hum Mutat. (2008) 29:803–8. doi: 10.1002/humu.20720 18409191

[7] Sarrauste de Menthière C Terrière S Pugnère D Ruiz M Demaille J Touitou I . INFEVERS: the Registry for FMF and hereditary inflammatory disorders mutations. Nucleic Acids Res. (2003) 31:282–5. doi: 10.1093/nar/gkg031 12520003 PMC165478

[8] Robinson PN Köhler S Bauer S Seelow D Horn D Mundlos S . The Human Phenotype Ontology: a tool for annotating and analyzing human hereditary disease. Am J Hum Genet. (2008) 83:610–5. doi: 10.1016/j.ajhg.2008.09.017 18950739 PMC2668030

[9] Haimel M Pazmandi J Heredia RJ Dmytrus J Bal SK Zoghi S . Curation and expansion of Human Phenotype Ontology for defined groups of inborn errors of immunity. J Allergy Clin Immunol. (2022) 149:369–78. doi: 10.1016/j.jaci.2021.04.033 33991581 PMC9346194

[10] Maassen W Legger G Kul Cinar O van Daele P Gattorno M Bader-Meunier B . Curation and expansion of the Human Phenotype Ontology for systemic autoinflammatory diseases improves phenotype-driven disease-matching. Front Immunol. (2023) 14:1215869. doi: 10.3389/fimmu.2023.1215869 37781402 PMC10536149

[11] Banerjee J Taroni JN Allaway RJ Prasad DV Guinney J Greene C . Machine learning in rare disease. Nat Methods. (2023) 20:803–14. doi: 10.1038/s41592-023-01886-z 37248386 PMC13293574

[12] Amorim AMB Orzeł U Caniceiro AB Rosário-Ferreira N Moreira IS . Artificial intelligence in rare diseases: toward clinical impact. Trends Pharmacol Sci. (2025) 46:1241–68. doi: 10.1016/j.tips.2025.10.010 41298147

[13] Decherchi S Pedrini E Mordenti M Cavalli A Sangiorgi L . Opportunities and challenges for machine learning in rare diseases. Front Med. (2021) 8. doi: 10.3389/fmed.2021.747612 34676229 PMC8523988

[14] McInnes L Healy J Melville J . UMAP: uniform manifold approximation and projection for dimension reduction. (2020) arXiv:1802.03426v3. doi: 10.48550/arXiv.1802.03426

[15] Gerritsma AM Sutera D Cantarini L Cattalini M Lachmann HJ Minden K . TNFRSF1A-pR92Q variant identifies a subset of patients more similar to systemic undifferentiated recurrent fever than TNF receptor-associated periodic syndrome. Clin Exp Rheumatol. (2023) 41:1998–2007. doi: 10.55563/clinexprheumatol/am4phc 37470237

[16] Ben-Chetrit E Touitou I . The significance of carrying MEFV variants in symptomatic and asymptomatic individuals. Clin Genet. (2024) 106:217–23. doi: 10.1111/cge.14566 38818540

[17] Rowczenio DM Iancu DS Trojer H Gilbertson JA Gillmore JD Wechalekar AD . Autosomal dominant familial Mediterranean fever in Northern European Caucasians associated with deletion of p.M694 residue-a case series and genetic exploration. Rheumatol (Oxford). (2017) 56:209–13. doi: 10.1093/rheumatology/kew058 27150194

[18] Sag E Imren G Walle V Aydin EA Bayindir Y Cam V . Transcriptome analysis of unmedicated heterozygous familial Mediterranean fever patients reveals a type I interferon signature driving increasing Pyrin expression. Ann Rheum Dis. (2025) 84:1928–37. doi: 10.1016/j.ard.2025.08.001 40883219

