# Supplementary material for: Machine learning–based validation of Human Phenotype Ontology (HPO) terms for systemic autoinflammatory diseases using the Eurofever Registry: the ODINO project
Source: Front Immunol. 2026 Oct 12;17:1936099. doi: 10.3389/fimmu.2026.1936099 (PMC13697011; doi:10.3389/fimmu.2026.1936099)
Supplement: Supplementary file 1 [file Supplementaryfile1.docx]

***Supplementary Material***

**SUPPLEMENTARY METHODS**

**Dataset preparation**

After harmonization of EF variables into HPO terms, non-informative “other” categories were removed and redundant mappings were addressed by collapsing selected pairs of highly overlapping symptoms into a single binary feature using a logical OR rule. Additionally, HPO terms that were not present in any patient were excluded from the analysis, resulting in a set of 168 binary HPO-derived clinical features describing disease manifestations. Demographic and clinical variables (age at onset, fever length	, gender	, and ancestry	) were appended, yielding a total of 172 input variables	.

In order to better exploit the hierarchical structure of the HPO, an extended version of the dataset was subsequently generated by incorporating ancestor terms. Specifically, for each annotated HPO term, its parent terms within the ontology hierarchy were added using the *ontologyIndex* R package, allowing the representation of both specific and more general phenotypic concepts. This approach was adopted to capture broader clinical patterns and improve phenotype comparability across patients.

Following this hierarchical expansion, the dataset included 429 HPO terms, together with the same set of additional clinical and demographic variables (age at onset, fever length	, gender	, and ancestry	), resulting in 433 predictors plus the diagnosis. As in the original dataset, the diagnosis was retained as the target variable for the supervised analysis.

Both dataset versions (with and without ancestors) were used for downstream supervised analyses in order to assess the impact of hierarchical phenotype enrichment on diagnostic performance. In all subsequent analyses, models trained on the dataset including HPO ancestor terms were considered as the primary reference, while models trained without hierarchical enrichment were used for sensitivity analyses.

The dataset, including 2,576 patients, was divided into a training set (70%, n=1,801) and a test set (30%, n=775), ensuring that the distribution of diagnoses was preserved across the two subsets. The test set was held out and used exclusively for the final evaluation of model performance.

All statistical analyses were performed using R version 4.4.3 and Python version 3.8.20.

Evaluation of HPO terms performance using machine learning models

A supervised ML framework was implemented to evaluate the diagnostic performance of HPO-based phenotypic representations. Several classification strategies and algorithms were systematically explored using the same preprocessing and evaluation pipeline.

Four ML classifiers were considered: Elastic Net logistic regression (EN), k-Nearest Neighbors (kNN), Random Forest (RF), and eXtreme Gradient Boosting (XGBoost). These models were selected to cover different methodological approaches, including linear, distance-based, and ensemble tree-based methods. Elastic Net models were implemented using the LogisticRegression class from the scikit-learn library, Random Forest using RandomForestClassifier, and kNN using KNeighborsClassifier (Pedregosa et al., 2011), while gradient boosting models were implemented using XGBClassifier from the XGBoost library (Chen & Guestrin, 2016).

Two different classification strategies were investigated. First, a multiclass classification setting was implemented, in which each patient was directly assigned to one of the diagnostic categories included in the study. Second, a one-vs-rest (OVR) approach was adopted. In this setting, a separate binary classifier was trained for each disease to distinguish patients affected by the target condition from all other diagnoses grouped together as “Other”. Both multiclass and OVR approaches were applied consistently across all evaluated ML models.

**Hyperparameter tuning and model evaluation**

Model training and hyperparameter tuning were performed exclusively on the training set (70%) using a nested 5-fold cross-validation (CV) framework with random shuffling:

- In the outer loop, the training set was divided into 5 folds. At each iteration, four folds were used for model training and hyperparameter selection, while the remaining fold served as a validation set to estimate model performance.
- Within each outer-loop training split, an inner 5-fold CV was performed to tune hyperparameters using grid search. In this step, candidate hyperparameter configurations were evaluated across the five inner folds and the configuration achieving the best average F2-score was selected.

The selected configuration was then refitted on the entire outer-loop training partition and evaluated on the corresponding outer validation fold.

This nested procedure was repeated across all outer folds to obtain an unbiased estimate of model performance.

Detailed performance metrics across folds and models are reported in **Supplementary Tables E**	**1-E**	**4**.

**Hyperparameter search space**

Hyperparameter tuning was performed using predefined grid search spaces for each algorithm.

For XGBoost models, the grid included the number of trees (n_estimators = 100, 500), maximum tree depth (max_depth = 3, 5, 7), learning rate (learning_rate = 0.3, 0.1), column subsampling rate (colsample_bytree = 0.5, 1.0), and minimum child weight (min_child_weight = 1, 5). This grid was designed to balance model expressiveness and computational efficiency, while limiting overfitting. These analyses were conducted using the XGBClassifier from the Python XG Boost library (Chen & Guestrin, 2016).

For Elastic Net logistic regression, the hyperparameter grid included a fixed inverse regularization strength (C = 0.1) and three values for the L1/L2 mixing parameter (l1_ratio = 0.2, 0.5, 0.8). For Random Forest, the number of trees (n_estimators = 100, 500), maximum tree depth (max_depth = 3, 5, 7), and minimum leaf size (min_samples_leaf = 1, 5) were explored.

For k-Nearest Neighbors, the number of neighbors (n_neighbors = 5, 11, 21), distance weighting scheme (uniform or distance), and distance metric (p = 1 or 2) were evaluated (Pedregosa et al., 2011).

These search spaces were designed to balance model expressiveness and computational efficiency while limiting the risk of overfitting.

For algorithms requiring feature scaling (Elastic Net and k-Nearest Neighbors), predictors were standardized using a standard scaler. Missing values were handled using median imputation within the training folds.

Class imbalance was addressed during model training using sample weights.

For multiclass models, class weights were computed according to the formula:

*w_c_ = N / (K · n_c_)*

where *N* is the total number of samples, *K* the number of classes, and *n_c_* the number of samples in class *c*.

For OVR classifiers, negative samples were assigned a weight of 1, whereas positive samples were weighted proportionally to the imbalance between negative and positive cases (n_neg_ / n_pos_).

This weighting scheme ensured that minority classes contributed proportionally to model training.

**Test set evaluation**

The test set (30%) was used exclusively for final model evaluation.

In the OVR framework, five disease-specific binary classifiers were trained (CAPS, FMF, MKD, PFAPA, and TRAPS). During the test phase, each patient was independently evaluated by all disease-specific classifiers, producing a set of disease-specific predicted probabilities.

Each binary classifier was first evaluated independently using the default probability threshold of 0.50.

To obtain a single disease prediction for each patient and enable direct comparison with the multiclass approach, the five disease-specific probabilities were subsequently normalized on a patient-by-patient basis. Specifically, each probability was divided by the sum of the five probabilities generated for that patient, resulting in normalized scores summing to 1. The disease with the highest normalized score was assigned as the final prediction. This normalization preserves the relative ranking of the original OVR probabilities.

Patient-level predicted probabilities obtained for each individual in the test set are provided in **Supplementary Tables E**	**5 and E**	**6**.

**Model interpretability**

Feature importance analyses were performed for models allowing direct interpretability.

For XGBoost models, feature importance was quantified using the gain metric, which reflects the average improvement in model performance contributed by a feature when used for splitting.

In addition, model interpretability was further investigated using SHapley Additive exPlanations (SHAP) [Lundberg & Lee, 2017], which quantify the contribution of each feature to individual model predictions. Disease-specific SHAP importance rankings are reported in **Supplementary Tables E**	**9-E**	**13**.

**Error analyses**

Error analyses were performed on the test set to investigate systematic patterns of misclassification, including subgroup-specific enrichments.

Furthermore, diagnostic performance was also evaluated considering the two most probable predicted diseases (top-2 predictions) in order to assess whether the correct diagnosis was captured among the highest-ranking model suggestions. Results of this analysis are reported in **Supplementary Tables E**	**7 and E**	**8**.

**Unsupervised phenotypic similarity analysis**

UMAP was used to visualize the phenotypic distribution of FMF patients carrying one versus two pathogenic *MEFV* mutations. To quantitatively assess group separability, the silhouette score was additionally calculated in the original feature space using the non-propagated HPO terms and the corresponding clinical/demographic variables. Continuous variables were standardized and categorical variables were one-hot encoded. The resulting silhouette score was 0.010.

**Development of web app to support clinical decision making**

Finally, in order to facilitate the practical use of the proposed model, we developed a web-based application implemented using Streamlit (https://streamlit.io/), which allows users to input patient-specific HPO terms and obtain disease probability predictions generated by the final XGBoost OVR model. Source code is available at https://github.com/Jackcava/hpo-dx.

**Supplementary tables**

**Supplementary Table 1.** EUROFEVER terms with an absolute HPO correspondence (in red, the EF redundant terms)

| **EUROFEVER TERMS** | **HPO CODE** | **DESCRIPTION** |
| --- | --- | --- |
| Muco-cutaneous manifestations | HP:0000951 | Abnormality of the skin |
| Musculoskeletal system | HP:0033127 | Abnormality of the musculo-skeletal system |
| Ocular manifestation | HP:0000478 | Abnormality of the eye |
| Gastrointestinal system | HP:0011024 | Abnormality of the GI tract |
| Lymphoid organs | HP:0100763 | Abnormality of the lymphatic system |
| Disease Course | HP:0011008 | Temporal pattern |
| Cardio-respiratory system | HP:0001626 | abnormality of the cardiovascular system |
| Neurological manifestations | HP:0000707 | Abnormalities of the nervous system |
| Genito - Urinary manifestations | hp:0000119 | Abnormality of the genito-urinary system |
| Constitutional symptoms | HP:0025142 | Constitutional symptom |
| Hematological alteration | HP:0001871 | Abnormality of blood and blood-forming tissues |
| Chest pain | HP:0100749 | Chest Pain |
| Dyspnoea | HP:0002094 | Dyspnea |
| Pericarditis | HP:0001701 | Pericarditis |
| Pleurisy | HP:0002102 | Pleuritis |
| Pneumonia - | HP:0011949 | Acute Infectious Pneumonia |
| Interstitial lung | HP:0006530 | Abnormal pulmonary interstitial morphology |
| Persistent cough | HP:0034315 | Chronic cough |
| Pulmonary vasculitis | HP:0034705 | Pulmonary vasculitis |
| Hemoptysis | HP:0002105 | Hemoptysis |
| Venous thrombosis | HP:0004936 | Venous thrombosis |
| Arterial thrombosis | HP:0004420 | Arterial thrombosis |
| Myocarditis | HP:0012819 | Myocarditis |
| Large vessel vasculitis | HP:0005310 | Large vessel vasculitis |
| Myocardial ischemia, infarction | HP:0001658 | Myocardial infarction |
| Cardiac hypertrophy | HP:0001639 | Hypertrophic cardiomyopathy |
| Arterial aneurysm/stenosis | HP:0100545  HP:0002617 | Arterial stenosis  Aneurysmal dilatation |
| Large vessel vasculopathy | HP:0005310 | Large vessel vasculitis |
| Medium/small vessels vasculitis | HP:6000658  HP:0011944 | Medium vessel vascuilitis  Small vessels vasculitis |
| Cardiomyopathy | HP:0001638 | Cardiomyopathy |
| Pulmonary fibrosis | HP:0002206 | Pulmonary fibrosis |
| Fever (>38) | HP:0001945 | fever |
| Low Grade fever (<38) | HP:0011134 | Low grade fever |
| Fatigue | HP:0012378 | Fatigue |
| Malaise | HP:0033834 | Malaise |
| Mood disorders | HP:0000712 | Emotional lability |
| Sensation of fever, chills without fever | HP:0025143 | Chills |
| Weight loss | HP:0001824 | Weight loss |
| Fever >38º C | HP:0001945 | Fever |
| Low grade fever <38º | HP:0011134 | Low-grade fever |
| Chills at fever onset | HP:0025143 | Chills |
| Triggers | HP:0025204 | Triggered by |
| Cold | HP:002526 | Triggered by cold |
| Emotional stress | HP:0025226 | Triggered by stress |
| Vaccination | HP:0025219 | Triggered by vaccination |
| Infection | HP:0033184 | Triggered by infection |
| Exercise | HP:0025377 | Triggered by exertion |
| Trauma | HP:0031135 | Triggered by physical trauma |
| Food | HP:0033793 | Triggered by food ingestion |
| Menstruation | HP:0025220 | Triggered by menstruation |
| Fatigue | HP:0012378 | Fatigue |
| Vomiting | HP:0002013 | Vomiting |
| Abdominal pain | HP:0002027 | Abdominal pain |
| Constipation | HP:0002019 | Constipation |
| Diarrhea | HP:0002014 | Diarrhea |
| Gastrointestinal ulcers | HP:0034274 | Gastrointestinal ulcer |
| GI bleeding | HP:0002239 | Gastrointestinal hemorrhage |
| Mesenteric aneurysm | HP:0011934 | Dilatation of mesenteric artery |
| Hepatitis | HP:0012115 | Hepatitis |
| Portal hypertension | HP:0001409 | Portal hypertension |
| Gastritis | HP:0005263 | Gastritis |
| Gut perforation | HP:0031368 | Intestinal perforation |
| Peritoneal adhesions | HP:0033134 | Abdominal adhesions |
| Intestinal occlusion/sub-occlusion | HP:0005214 | Intestinal obstruction |
| Gonadal pain without proven ischaemia | HP:0033839 | Testicular pain |
| Gonadal infarction | HP:0033403 | testicular ischemia |
| Epididymitis | HP:0000031 | Epididymitis |
| Essential hypertension | HP:0000822 | Hypertension |
| Renovascular hypertension | HP:0100817 | Renovascular hypertension |
| Renal infarction | HP:0032618 | kidney infarction |
| Renal aneurysm | HP:0033261 | Renal artery aneurysm |
| Kidney amyloidosis | HP:0001917 | Renal amyloidosis |
| Glomerulonephritis | HP:0000099 | Glomerulonephritis |
| Glomeruloscerosis | HP:0000096 | Glomerular sclerosis |
| Pancytopenia | HP:0001876 | Pancytopenia |
| Leucopenia | HP:0001882 | Leukopenia |
| Thrombocytopenia | HP:0001873 | Thrombocytopenia |
| Aplastic anemia | HP:0001915 | Aplastic anemia |
| Neutropenia | HP:0001875 | Neutropenia |
| Lymphopenia | HP:0001888 | Lymphopenia |
| Hypogammaglobulinemia | HP:0004313 | Decreased circulating antibody level |
| Hypercoagulability | HP:0100724 | Hypercoagulability |
| Recurrent infections | HP:0002719 | Recurrent infections |
| Severe infections (hospitalization) | HP:0032169 | Severe infection |
| Opportunistic infections (bacterial, viral, elimintic, other) | HP:0031690 | Opportunistic infection |
| Leukemia | HP:0001909 | Leukemia |
| Hydiopatic myelofibrosis | HP:0011974 | Myelofibrosis |
| Generalized lymphnodes enlargement | HP:0008940 | Generalized lymphadenopathy |
| Enlarged cervical lymphnodes | HP:0025289 | Cervical lymphadenopathy |
| Hepatomegaly | HP:0002240 | Hepatomegaly |
| Splenomegaly | HP:0001744 | Splenomegaly |
| Splenic infarct | HP:0034336 | Splenic infarction |
| Parotitis | HP:0011850 | Parotitis |
| Aphthous stomatitis | HP:0000155 | Oral ulcers |
| Exudative pharyngitis | HP:0034035 | Pharyngeal exudate |
| Maculo-papular rash | HP:0040186 | Maculopapular exanthema |
| Urticarial rash | HP:0001025 | Urticaria |
| Palpable purpura | HP:0031363 | Palpable purpura |
| Erysipelas-like erythema | HP:0001055 | Erysipelas |
| Pseudo-folliculitis | HP:0025084 | folliculitis |
| Acne | HP:0001061 | Acne |
| Psoriasis | HP:0003765 | Psoriasiform dermatitis |
| Ulcers at genitalia | HP:0003249 | Genital ulcers |
| Pyoderma gangrenosum | HP:0025452 | Pyoderma gangrenosum |
| Palmo-plantar pustulosis | HP:0100847 | Palmoplantar pustulosis |
| Erythema nodosum EN | HP:0012219 | Erythema nodosum |
| Other Panniculitis non-EN | HP:0012490 | Panniculitis |
| Positive pathergy test | HP:0025532 | Positive pathergy test |
| Erythematous/violaceous plaques | HP:0025474 | Erythematous plaque |
| Lipodystrophy | HP:0009125 | lipodystrophy |
| Eyelid violacoeus swelling | HP:0100540 | Palpebral edema |
| Cutaneous ulceration | HP:0200042 | Skin ulcer |
| Alopecia | HP:0001596 | Alopecia |
| Livedo racemosa/reticularis | HP:0033260  [HP:0033505](https://hpo.jax.org/browse/term/HP:0033505) | Livedo racemosa  Livedo reticularis |
| Eczema | HP:0000964 | Eczema |
| Subcutaneous nodules | HP:0001482 | Subcutaneous nodule |
| Photosensitivity | HP:0000992 | Cutaneous photosensitivity |
| Raynaud’s phenomenon | HP:0030880 | Raynaud phenomenon |
| Chilblains | HP:0009710 | Chilblains |
| Nail distrophy | HP:0008404 | Nail dystrophy |
| Arthralgia | HP:0002829 | Arthralgia |
| Myalgia | HP:0003326 | Myalgia |
| Muscle weakness | HP:0001324 | Muscle weakness |
| Myositis | HP:0100614 | Myositis |
| Fasciitis | HP:0100537 | Fascitiis |
| Bone pain | HP:0002653 | Bone pain |
| Osteomyelitis - infectious | HP:0002754 | Osteomyelitis |
| Oligoarthritis - ≤4 joints | HP:0040313 | Oligoarthritis |
| Polyarthritis - ≥5 joints | HP:0005764 | Polyarticular arthritis |
| Dactylitis | HP:0031090 | Finger dactylitis |
| Flexion contractures | HP:0001371 | Flexion contracture |
| Bone alteration | HP:0003330 | Abnormal bone structure |
| Patellar overgrowth | HP:0033308 | Patellar overgrowth |
| Frontal bossing | HP:000207 | Frontal bossing |
| Clubbing | HP:0001217 | Clubbing |
| Camtpodactily | HP:0012385 | Camptodactily |
| Osteolytic lesions | HP:0002797 | Osteolysis |
| Hyperostosis | HP:0100774 | Hyperostosis |
| Osteoporosis | HP:0000939 | Osteoporosis |
| Muscular atrophy | HP:0003202 | Skeletal muscle atrophy |
| Headache - anytime | HP:0002315 | Headache |
| Optic neuritis | HP:0100653 | Optic neuritis |
| Seizures | HP:0001250 | Seizure |
| Cranial nerve palsy | HP:0006824 | Cranial nerve paralysis |
| Vertigo | HP:0002321 | Vertigo |
| Ataxia | HP:0001251 | Ataxia |
| Peripheral neuritis | HP:0009830 | Peripheral neuropathy |
| Transient Ischemic Attack - TIA | HP:0002326 | Transient ischemic attack |
| Stroke | HP:0001297 | Stroke |
| Intracranial hemorrhage | HP:0002170 | Intracranial hemorrhage |
| Hydrocephalus | HP:0000238 | Hydrocephalus |
| Neurosensorial hearing loss | HP:0000407 | Sensorineural hearing impairment |
| Peripheral neuropathy | HP:0009830 | Peripheral neuropathy |
| Tetra/paraplegia | HP:0002445  HP:0010550 | Tetraplegia  Paraplegia |
| Mental retardation | HP:0001249 | Intellectual disability |
| Periorbital oedema | HP:0100539 | Periorbital edema |
| Conjunctivitis | HP:0000509 | Conjunctivitis |
| Uveitis anterior | HP:0012122 | Anterior uveitis |
| Uveitis posterior | HP:0012123 | Posterior uveitis |
| Keratitits | HP:0000491 | Keratitis |
| Scleritis | HP:0100532 | Scleritis |
| Episcleritis | HP:0100534 | Episcleritis |
| Lacrymal gland involvement | HP:0000620 | Dacryocystitis |
| Papilledema | HP:0001085 | Papilledema |
| Papillitis | HP:0001085 | Papilledema |
| Optic nerve atrophy | HP:0000648 | Optic atrophy |
| Retinal vasculitis | HP:0025188 | Retinal vasculitis |
| Cataract | HP:0000518 | Cataract |
| Glaucoma | HP:0000501 | Glaucoma |
| Band keratopathy | HP:0000585 | Band keratopathy |
| Optic nerve atrophy | HP:0000648 | Optic atrophy |
| Impaired vision | HP:0000505 | Impaired vision |
| Blindness | HP:0000618 | Blindness |
| AA- Amyloidosis | HP:0011034 | Amyloidosis |
| Macrophage activation syndrome | [HP:0012156](https://hpo.jax.org/app/browse/term/HP:0012156) | hemophagocytosis |
| Infertility | HP:0000789 | Infertility |
| Tumors | HP:0002664 | Neoplasm |
| Failure to thrive | HP:0001508 | Failure to thrive |
| Gender / height related hypertension | HP:0000822 | Hypertension |
| CRP | HP:0032436 | Abnormal circulating C-reactive protein concentration |
| ESR | HP:0025021 | Abnormal erythrocyte sedimentation rate |
| Serum amyloid A | HP:0033332 | Elevated circulating amyloid A |
| Urine mevalonic acid measurement | HP:0032638 | Elevated urine mevalonic acid level |
| WBC | HP:0001974 | Leukocytosis |
|  |  | |

**Supplementary Table 2.** Eurofever terms with partial HPO correspondence

| EUROFEVER TERMS | HPO 1 | DESCRIPTION | | | HPO 2 | | DESCRIPTION |
| --- | --- | --- | --- | --- | --- | --- | --- |
| Lymphatic dysplasias | HP:0100766 | Abnormal Lymphatic [HP:0003759](https://hpo.jax.org/browse/term/HP:0003759) Hypoplasia of lymphatic vessel morphology vessels | | | | | |
| Pattern of frequency | HP:0040279 | Frequency | | | | HP:0025304 | Periodic |
| Anal/perianal ulcers | HP:0012390 | Anal fissure | | | | |  |
| Aseptic Peritonitis | HP:0002586 | Peritonitis | | | | |  |
| Mesenteric infarct | HP:0005244 | Gastrointestinal infarctions | | | | HP:0033404 | Intestinal ischemia |
| Intestinal amyloidosis | HP:0011034 | Amyloidosis | | | | |  |
| Urethritis/ cistitis | HP:0500006 | Urethritis | | | | HP:0100577 | Urinary bladder inflammation; Cystitis of the urinary bladder |
| Necrotising pneumonia | HP:0033243 | Pulmonary necrosis | | | |  |  |
| Erythematous pharyngitis | HP:0025439 | Pharyngitis | | | | |  |
| Migratory rash | HP:0033622 | Migratory erythematous plaque | | | | | |
| Papulopustular lesions | HP:0033605 | Pustular rash | | | | |  |
| Ichthyosiform rash | HP:0008064 | Ichthyosis | | | | |  |
| Digital/peripheral ischaemia (gangrenous) | HP:0033401 | Digital ischemia | | | | |  |
| Digital/peripheral ischaemia (non-gangrenous) | HP:0033402 | Digital ischemia | | | | |  |
| Discoid skin lesion | HP:0033120 | Nummular eczema | | | | HP:0007417 | Discoid lupus rash |
| Monoarthritis | HP:0001369 | Arthritis | | | |  |  |
| Tenosynovitis | HP:0025230 | Tendonitis | | | | HP:0100769 | Synovitis |
| Digit(s) amputation | HP:0007460 | Autoamputation of digits | | | | | |
| Limb amputation | HP:0001868 | Autoamputation of foot | | | | | |
| Bone Deformity | HP:0002814 | Abnormality of lower limbs | | | | | |
| Bone erosions | HP:0003106 | Subperiosteal bone erosions | | | | | |
| Aseptic meningitis | HP:0001287 | Meningitis | | | | |  |
| Cranial neuropathy | HP:0006824 | Cranial nerve palsy | | | | |  |
| Headache (morning) | HP:0002315 | Headache | | | | |  |
| Culture proven severe infections | HP:0032169 | Severe infection | | | | |  |
| Death | HP:0011420 | Age of death | | | | |  |
| Age at onset | | | HP:0003623  HP:0410280  HP:0003581 | Neonatal  Pediatric  Adult | | | |

**Supplementary Table 3.** EF TERMS without correspondence in the HPO. Specifics of why an adequate correspondent term is not present are given in the second column when appropriate

| Gender |  |
| --- | --- |
| Ancestry* |  |
| Mean duration of episodes (fever length) |  |
| Number of episodes/year |  |
| Seasonal changes |  |
| Travel (triggered by) |  |
| Intestinal vasculitis | The term vasculitis in general is present, with specificity about skin, muscle, cerebral, nasal, retinal and renal vasculitis |
| Painful lymph nodes | Lymphadenopathy HP:0002716 present, but no indication regarding any pain |
| Osteitis - non-infectious | Only the term “osteomyelitis” **HP:0002754** present in HPO and labeled as infectious osteomyelitis, even if the term is associated to CRMO |
| Cerebellar syndrome |  |
| Periorbital pain | Only periorbital oedema present |
| Periorbital erythema | “ |
| Coronaries' arteritis | No terms referring to coronary vasculitis |

* Reported in EF as: African, (African descent), Arctic (Siberian, Eskimo), Caucasian (European), Caucasian (Indian), Caucasian (Middle East), Caucasian (North African, Other), Hispanic (for US), Indigenous Australian, Mixed Ethnicity, Native American, North East Asian (Mongol, Tibetan, Korean Japanese, etc), Pacific (Polynesian, Micronesian, etc), South East Asian (Chinese, Thai, Malay, Filipino, etc), Other Ethnicity,

**Supplementary Table 4.** XGBoost performance metrics for each disease in the final combined OVR single-label classification	on the held-out test set (30%	).

| **Disease** | F2-score | Bal. acc. | MCC | Recall | Precision |
| --- | --- | --- | --- | --- | --- |
| **CAPS** | 0.8209 | 0.8964 | 0.8156 | 0.8105 | 0.8652 |
| **FMF** | 0.8802 | 0.9011 | 0.8056 | 0.8719 | 0.9152 |
| **MKD** | 0.5556 | 0.7611 | 0.5223 | 0.5556 | 0.5556 |
| **PFAPA** | 0.8745 | 0.9095 | 0.7787 | 0.9043 | 0.7727 |
| **TRAPS** | 0.5699 | 0.7598 | 0.5485 | 0.557 | 0.6286 |

**Supplementary Figures**

**Supplementary Figure 1. Cross-validation comparison of ML models.**

Distribution of F2-scores obtained during nested cross-validation for the evaluated machine learning algorithms under multiclass and OVR classification strategies across diseases.

**Supplementary Figure 2. SHAP beeswarm plots illustrating feature contributions to disease-specific XGBoost classifiers.**

Each point represents an individual patient and its horizontal position corresponds to the SHAP value of that feature. Colors indicate the feature value (blue = low, red = high). For binary HPO features, high values correspond to the presence (1) and low values to the absence (0) of the phenotypic term.
